# Minicircles for Investigating and Treating Arthritic Diseases

**DOI:** 10.3390/pharmaceutics13050736

**Published:** 2021-05-17

**Authors:** Yeri Alice Rim, Yoojun Nam, Narae Park, Ji Hyeon Ju

**Affiliations:** 1 Catholic iPSC Research Center, College of Medicine, The Catholic University of Korea, Seoul 06591, Korea; llyerill0114@gmail.com (Y.A.R.); givingtreemax@gmail.com (Y.N.); narae5322@gmail.com (N.P.); 2 Division of Rheumatology, Department of Internal Medicine, Seoul St. Mary’s Hospital, Institute of Medical Science, College of Medicine, The Catholic University of Korea, Seoul 06591, Korea

**Keywords:** minicircle, cartilage, osteoarthritis, rheumatoid arthritis, gene delivery, gene therapy

## Abstract

Gene delivery systems have become an essential component of research and the development of therapeutics for various diseases. Minicircles are non-viral vectors with promising characteristics for application in a variety of fields. With their minimal size, minicircles exhibit relatively high safety and efficient delivery of genes of interest into cells. Cartilage tissue lacks the natural ability to heal, making it difficult to treat osteoarthritis (OA) and rheumatoid arthritis (RA), which are the two main types of joint-related disease. Although both OA and RA affect the joint, RA is an autoimmune disease, while OA is a degenerative joint condition. Gene transfer using minicircles has also been used in many studies regarding cartilage and its diseased conditions. In this review, we summarize the cartilage-, OA-, and RA-based studies that have used minicircles as the gene delivery system.

## 1. Introduction

Gene therapy is defined as the delivery of vectors encoding a gene of interest into cells, aiming to modify endogenous gene expression, and is used in research and the development of treatments [1,2]. Bacterial plasmid DNA is currently widely used in various fields with their application ranging from “the bench to the bed”. The safe and efficient expression of gene delivery is promising for both in vitro and in vivo use. While it is believed to be a relatively safe gene transfer strategy, commercial plasmid DNA vectors contain bacterial sequences that may induce potential immune responses [3]. In addition, several critical sequences of these vectors, such as antibiotic resistance markers, increase plasmid size, which leads to decreased transfection efficacy into host cells. To overcome these issues, minicircles have been suggested as an alternative [4]. The relatively small size of minicircles compared to that of other commercial vectors has been achieved by eliminating the bacterial backbones and other non-critical transcription units, which enables a higher level of expression of the gene of interest both in vitro and in vivo [5]. 

The limited ability of articular cartilage (AC) tissue to self-repair strongly requires the development of an effective treatment or repair therapy, along with extensive investigational research [6]. Various approaches have been attempted in the investigation of damaged AC, including the search for the most effective treatment method [7]. The development of DNA-based drugs or therapeutics for arthritic diseases is an appealing strategy, with the target protein of interest being produced through cell transcriptional processes. Intra-articular gene therapy was initially developed to overcome the pharmacokinetic barriers associated with joints [7]. The successful transfer of a gene-of-interest in the joint may induce the endogenous synthesis of therapeutic gene products by the residing cells. For this purpose, minicircles have been suggested as a suitable tool and have been used in various studies dealing with arthritic diseases, such as osteoarthritis (OA) and rheumatoid arthritis (RA). 

In this review, we focus on OA- and RA-related studies that used minicircles. We provide an overview of the value of minicircles in studies of arthritis and various approaches and summarize the relative findings of (1) basic research, (2) disease modeling, and (3) the development of therapeutics for the recovery of the joint cartilage.

## 2. AC and Arthritic Diseases

AC is an avascular and aneural connective tissue that is necessary for the proper movement of skeletal bones [8]. Covering the end of the bone with cartilage protects it from fracture that can be caused by external forces. Cartilage tissue is usually classified into three types, hyaline, fibrous, and elastic cartilage, with AC being hyaline cartilage that exists in joints [9]. The avascular and aneural characteristics of AC make it difficult to transfer nutrients to damaged cells, making AC a tissue that lacks the ability of self-repair. 

### 2.1. OA and RA 

OA and RA are the most common and disabling forms of joint disease. While RA is an autoimmune disease, OA is usually thought to be caused by mechanical factors. However, OA is now generally accepted to be an inflammatory disease as well [10]. RA and OA share symptoms, such as joint inflammation and cartilage degeneration, which lead to extensive cartilage damage and the induction of the disabilities. RA fibroblast-like synoviocytes (FLSs) show excessive proliferation, migration, and invasion, playing a critical role in RA pathogenesis [11]. Synovitis caused by this phenomenon leads to cartilage destruction and joint destruction. In this review, we primarily focus on the use of minicircles in research related to OA and RA.

### 2.2. Current Treatment Strategies for OA and RA

Both OA and RA do not yet have an ideal therapy that can fully cure the disease [12]. In the case of RA, the overall goal is to relieve pain and decrease inflammation [13]. As the first-line management of RA, nonsteroidal anti-inflammatory drugs are generally prescribed to RA patients. The second-line treatment of RA is to delay the progression of joint destruction that leads to deformity. Disease modifying anti-rheumatic drugs such as methotrexate are commonly used [14].

Drugs are also used to treat OA as well; however, cartilage protection and regeneration is commonly attempted to treat OA [15]. Autologous chondrocyte implantation (ACI) is commonly performed on patients to induce cartilage regeneration. However, it is difficult to maintain the characteristics of chondrocytes under culture, making this use of this practice continuously for treatment challenging. Currently other cell types such as mesenchymal stem cells and cartilage-tissue differentiated from pluripotent stem cells are suggested as an alternative [15,16,17].

## 3. Minicircles

The main characteristics of plasmid vectors that directly affect their transfection efficiency are (1) vector size, (2) plasmid topology, and (3) vector DNA content [18]. Smaller vectors with supercoiled isoforms are most efficient at entering the cells [2]. Minicircles are non-viral DNA vectors derived from parental plasmids. Two recombination sites exist at each end of the bacterial DNA backbone, which are removed through integrase-mediated recombination [4]. Elimination of the prokaryotic backbone sequences during the production of minicircles, such as the bacterial origin of replication and the antibiotic resistance gene, enables the plasmid vector to have a relatively small size [19]. Due to this characteristic, minicircles have higher transfection efficiencies, induce higher levels of transgene expression, and exhibit improved safety compared to that of traditional plasmid vectors. When delivered by hydrodynamic intravenous injections, minicircles induce immediate hepatic transgene expression in vivo [4]. Minicircles are currently being widely used in various applications, ranging from basic research to the manufacturing of advanced gene-based vaccines and therapeutic transgenes.

### 3.1. Minicircle Generation

In minicircle production achieved in *Escheichia coli*, the minicircle containing the therapeutic gene sequence is obtained from the parental plasmid using site specific recombination and the bacterial DNA is discarded [20]. The removal of problematic bacterial sequences was first reported by Darquet et al. [21]. The production rate and purification process has been an important issue during the minicircle production process [22]. Using various inducible recombinases, the development and enhancement of the minicircle generation process has focused on increasing recombination efficiency and the minimization of the induction of multimeric forms [4,21,22,23,24]. Plasmids require a bacterial origin of replication (*ori*) for reproduction in the host. After the production process, minicircles *ori* is eliminated, therefore, minicircles are no longer able to replicate and not considered as plasmids. 

### 3.2. Intracellular Felivery of Minicircles

Minicircles have been delivered using various methods in previous studies. While the conventional cationic liposime method was commonly used in early stages of research using minicircles, low transfection efficiency was an issue that needed to be overcome. Using electroporation technology, the transfection rate was highly improved. Non-viral gene delivery is reported to be low in the case of MSCs [25]. Minicircle delivery using the cationic liposome method in MSCs produced disappointingly low results of under 5% of the total cells [26]. Using nucleofection, minicircle transfection rates increased to up to 25–35% [27]. Notably, minicircle microporation showed the highest transfection efficiency, reaching up to 63–66%, and the best efficiency under low voltage and a single pulse (990 V, 40 ms, one pulse) [26].

## 4. Application of Minicircles in Arthritic Disease Research

**Figure 1:**
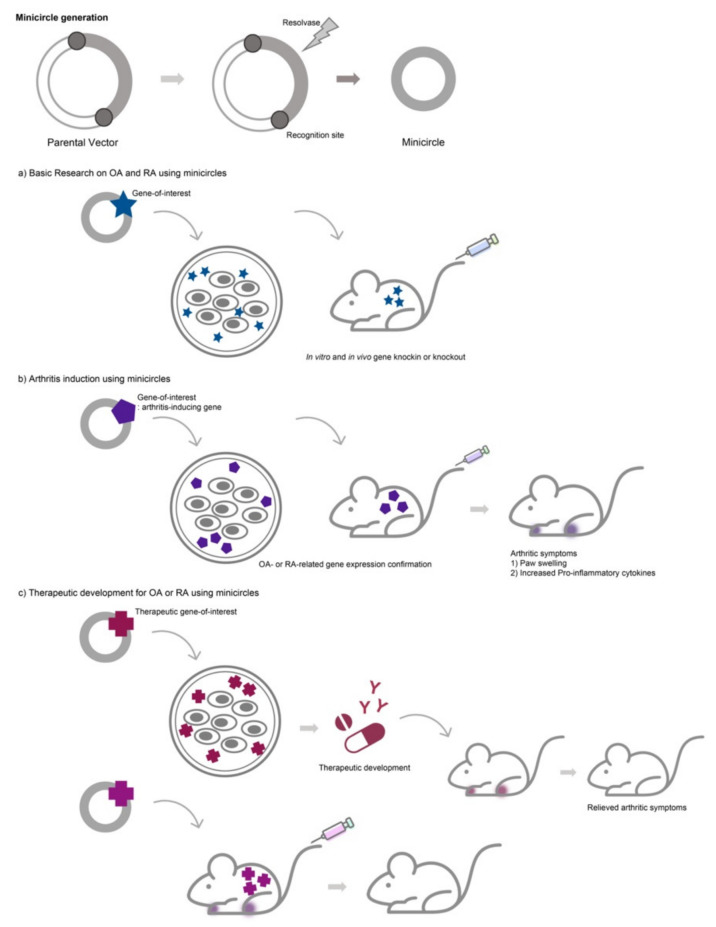
General application of minicircles for studies on arthritic diseases. Minicircles are generated from parental vectors. Studies using minicircles on arthritic diseases can be categorized as (**a**) in vitro and in vivo basic research on arthritic diseases, (**b**) inducing arthritis using minicircles for in vitro and in vivo disease modeling, and (**c**) gene therapy for treating arthritic diseases. Minicircles can be used directly as a tool for gene therapy in some cases or as delivery systems into cells to generate cell-based therapeutics for arthritis.

Minicircles have been applied in several studies related to arthritic diseases using different approaches. The purpose of the studies can be divided into three general groups: (1) basic research on arthritis, (2) arthritis induction, and (3) gene therapy and cartilage regeneration (Figure 1). 

### 4.1. Minicircles for Research on Arthritic Disease 

Two studies performed pre-clinical research on arthritis using minicircles (Table 1). Chao et al. confirmed the therapeutic effect of protein C17 (C17) (also known as cytokine-like protein 1 (Cytl1), C4orf4, and UNQ1942/PRO4425) in mice with collagen antibody-induced arthritis (CAIA) [28]. C17 is expressed in human bone marrow-derived or cord blood-derived CD34 positive cells, as well as in human cartilage explants, and has been reported to promote chondrogenic differentiation [29,30]. Chao et al. hypothesize that C17 is an unrecognized member of the interleukin (IL)-2 family and believe it may be related to the immune regulatory effects in arthritis. On day 35 post injection of C17-encoding minicircles into CAIA mice, hepatic expression of C17 mRNA and protein were confirmed. While the expression of C17 did not induce inflammation in normal mice, instead successfully delaying disease development and reducing swelling in the hind paws of the mice. Histological analysis showed that the minicircle-encoded C17 protected cartilage from damage. An analysis of expression in the hind paws of mice injected with C17-encoding minicircles revealed reduced gene expression related to pathologic tissue remodeling of joints and bones, including IL-1β and IL-6, along with impacting tumor necrosis factor α (TNFα), interferon γ (IFNγ), and IL-17 to lesser degrees.

The transfection rate of minicircles has been determined and compared in canine, equine, and rodent adipose-derived mesenchymal stem cells (MSCs) [19]. Due to their small size, canine MSCs exhibit comparably high transfection rates of minicircles compared to rodent MSCs; however, equine MSCs demonstrate a relatively low transfection rate. SOX9 is the most critical transcription factor related to effective chondrogenesis [31]. The overexpression of SOX9 has been attempted in canine MSCs and when SOX9-encoding minicircles are transfected into canine MSCs, increased expression of SOX9 protein is observed in the cell nucleus and cytoplasm [19]. The investigators of this study concluded that minicircles have the potential for use in gene therapy of non-rodent animal models for cartilage treatment.

In 2018, Choi et al. confirmed the effect of Kruppel-like factor 4 (KLF4) in rheumatoid arthritis (RA) [32]. KLF4 is a well-known factor related to cell survival, proliferation, and differentiation, and its expression has been confirmed to increase in the synovial tissue of patients with RA [33]. Choi and colleagues induced KLF4 overexpression in mice with collagen-induced arthritis (CIA) using minicircles. Three injections of KLF4-expressing minicircles near the first CIA induction induced severe arthritic symptoms with both the front and hind limbs becoming swollen compared to those of the mice injected with a mock minicircle. It was subsequently concluded that KLF4 regulates the proliferation and apoptosis of fibroblast-like synoviocytes (FLSs) in RA and its role in modifying the expression of matrix metalloproteinases (MMPs) MMP2, MMP12, and MMP13 and proinflammatory cytokines such as B-cell lymphoma 2 (BCL2), IL-6, and IFNγ was suggested.

### 4.2. Arthritis Induction Using Minicircles 

Using minicircles to induce arthritis has been attempted (Table 2). IL-23 is a pro-inflammatory cytokine that differentiates T helper 17 (Th17) cells. It has been reported that IL-23 transgenic mice die prematurely due to multiple organ inflammation, which limits studies on the effect of IL23 in arthritis and other diseases [34]. This led Adamopoulos and colleagues to evaluate the pathology in joints of mice overexpressing IL-23 using minicircle vectors [35]. The injection of an IL-23-expressing minicircle induced chronic swelling in the paws of the mice and serum IL-23 was detected for up to 90 days post injection, which was the latest time point evaluated. Bone marrow hyperplasia was observed 30 days after injection and an extensive loss of the subchondral lining was also observed, along with hyperplasia, inflammation, and pannus erosion. Th17 cells are characterized by the production of IL-17A, which induces arthritis progression and the production of RANKL, leading to osteoclast formation. Increased serum levels of pro-inflammatory cytokines including TNF, IL-6, IL-17A, IL-17F, IL-21, IL-22, IFNγ, and granulocyte-macrophage colony-stimulating factor (GM-CSF) are detected in late-stage (day 28) arthritic mice injected with IL-23 minicircles. Along with bone loss in the joints, bone mineral density is systemically decreased in the whole body of mice, which is confirmed to be correlated with an elevation in the serum levels of the osteoclast-associated marker TRAP-5b. To investigate the mechanism of IL-23-induced arthritis initiation, Adamopoulos et al. also measured IL-23 levels in the serum of early-stage (day 11) arthritic mice. Expression levels of IL-17A, IL-22, and IL-23 were observed to be significantly increased compared to that of control mice, while other cytokines, such as soluble RANKL, GM-CSF, IFNγ, IL-4, IL-6, and IL-21, were not consistently significantly different. However, IL-17A delivered using minicircles failed to induce paw inflammation, suggesting that IL-17A is incapable of initiating arthritis. Because IL-23 induces TNF expression and TNF transgenic mice developed chronic arthritis, these investigators attempted to identify the mechanism for IL-23-induced arthritis with regards to IL-17A, TNF, and CD4+ T cells. Blocking IL-17A or TNF along with the depletion of CD4+ T cells produced protective effects against the development of arthritis with modulation of TRAP5b. These data suggest that arthritis induction by IL-23-expressing minicircles is at least partly dependent on IL-17 and TNF, along with IL-17A-producing CD4+ T cells. This study by Adamopoulos et al. suggests IL-23 may be a suitable target to treat arthritic bone destruction. It is also one of the only studies in which arthritis has been induced using minicircle vectors.

### 4.3. Gene Therapy and Cartilage Regeneration using Minicircles 

Gene therapy has been attempted using minicircles in arthritic animal models (Table 3). Our group has published results from several studies confirming the therapeutic effects of minicircles [25,36,37,38,39]. Synthetic biologics have become the most widely used products in clinics; however, the high cost of these drugs is a major shortcoming, which is one reason we have attempted to generate drugs using minicircles. This concept was first attempted using minicircles encoding tocilizumab and etanercept [38]. Tocilizumab is an anti-IL-6 receptor (IL-6R) antibody that targets IL-6 signaling, while etanercept is a conjoined structure of the p75 region of TNF receptor 2 (TNFR2) and Fc portion of immunoglobulin that targets TNFα to treat patients with RA. The expression of both proteins from minicircles was confirmed by the transfection of HEK293T cells. The supernatant of the transfected cells inhibits the migration of RA-FLSs, which was confirmed by results from a scratch assay. When the minicircles encoding the drugs were injected into CIA mice, the arthritic symptoms were reduced and anti-IL-6R and soluble TNFR2 could be detected in the mouse serum. Immunohistochemical staining of liver tissue and the joints for green fluorescence protein (GFP) confirmed the presence and expression of the minicircles in the tissues of treated mice.

Abatacept, which is also known as cytotoxic T lymphocyte-associated antigen 4 immunoglobulin fusion protein (CTLA4Ig), is a co-stimulation inhibitor against B7/CD28 and is used to treat patients with autoimmune diseases, including RA. To resolve the issue of multiple injections being required for treatment and the high cost of synthetic protein drugs, we attempted to induce CTLA4Ig expression in vivo using minicircles [36]. Mock and parental vectors were injected into CIA mice, along with minicircles encoding CTLA4Ig (mcCTLA4Ig). While the mock and parental vectors failed to suppress the development of arthritis in the CIA mice, mcCTLA4Ig successfully reduced paw swelling. Furthermore, cartilage destruction was inhibited in the joints of the mice injected with mcCTLA4Ig at levels comparable to that of abatacept injections. The T-cell population was confirmed in mice, since abatacept is reported to increase the number of Forkhead box P3 (Foxp3)-positive T cells and inhibit the generation of Th17 cells. Splenocytes isolated from the spleens of mcCTLA4Ig-injected mice showed an increased number of Foxp3+ T cells and a reduced number of Th17 cells. Monocytes isolated from mcCTLA4Ig-injected mice also showed lower levels of osteoclast formation ex vivo. CTLA4Ig secreted from minicircles successfully reduced the in vitro osteoclast differentiation of monocytes in a dose-dependent manner.

As TNFα is a critical proinflammatory cytokine in RA, it is one of the main factors targeted to treat the disease. Because etanercept is one of the most successful biological drugs against TNFα, Park et al. attempted to produce and deliver this drug using minicircle-transfected MSCs (mcTNFR2MSCs) [25]. These investigators demonstrated that TNFR2-encoding minicircles can be successfully transfected into MSCs and that they do not alter the main characteristics of the cells, including the high expression of CD73 and CD105 and the low expression of CD34 and CD45. The compound induced by the minicircles reduced the migration of RA-FLSs and osteoclast differentiation in vitro. Furthermore, the drug-containing minicircle relieved cartilage destruction in the CIA mice, but mcTNFR2MSCs exhibited a higher therapeutic effect. Using a dual promoter vector, GFP was expressed to confirm the successful delivery of minicircles. Staining for GFP confirmed the expression of the minicircles, which was maintained until day 30 in the spleen and joints of the mice injected with mcTNFR2MSCs.

Patients with RA often do not respond to monospecific drugs. Therefore, our group has also generated an etanercept/tocilizumab hybrid form of drug to treat RA, which targets IL-6 signaling [39]. The developed the hybrid drug consists of soluble TNFR2 and anti-IL-6R antibodies and is referred to as a dual target agent (DTA). The DTA successfully blocked TNFα and IL-6 signaling in RA-FLSs by blocking the expression of pro-inflammatory factors, such as vascular endothelial growth factor (VEGF), GM-CSF, and MMP-3. When directly injected into the CIA mice intravenously, the destruction of cartilage was inhibited by the DTA secreted from the minicircles and both the soluble TNFR2 and anti-IL-6R antibodies could be detected in the serum.

SOX5 and SOX6 are co-factors of SOX9 and are critical elements for the integrity of chondrogenesis [40]. The delivery of these three genes into bone marrow-derived MSCs and adipose-derived stem cells (ADSCs) enhances chondrogenesis in vitro and arrests the progression of OA in animal models [41,42]. Using minicircles, Jeong et al. delivered SOX9, SOX6, and RNA interference (RNAi) against angiopoietin-like protein 4 (ANGPTL4) to enhance chondrogenic differentiation and suppress OA progression [43]. ANGPTL4 is involved in inflammation, tumorigenesis, and angiogenesis in various tissues and has also been confirmed in chondrocytes. While ANGPTL4 is reported to enhance cell hypoxia and upregulate the expression of MMPs, the expression of ANGPTL4 is higher in RA cartilage and OA cartilage compared to that in normal cartilage [44,45]. ANGPTLA4 RNAi knockdown successfully inhibits the expression of MMP13, MMP1, and MMP3 in chondrogenically differentiated MSCs [46]. Jeong et al. excluded SOX5 since recent studies have shown that SOX5 and SOX6 have similar functions and SOX6 is able to act alone as a co-factor for SOX9 [47]. These investigators also attempted to form nanoparticles containing these vectors using dexamethasone (DEX)-cross-linked polyethyleneimine (PEI) as a gene delivery system. They demonstrated that the delivered SOX9/SOX6/ANGPTLA4 RNAi-harboring minicircles (MC_9/6/shANG) successfully increased the expression of SOX9 and SOX6, while decreasing ANGPTL4 expression in ADSCs. Cells transfected with MC_9/6/shANG exhibited the highest chondrogenic efficacy during the formation of chondrogenic pellets and the greatest ability to reduce arthritis progression in surgically induced OA rats. Furthermore, the delivery of ANGPTLA4 RNAi successfully inhibited the expression of MMP3 and MMP13 in transfected cells, leading to a decreased expression of MMP13 and COX-2 in OA rat synovial fluid. While no comparison was performed between the nanoparticle delivery system and that of other methods, such as lipofectamine, this approach is expected to show an increased delivery of minicircles in ADSCs.

Recombinant growth factors are widely used for in vitro cellular differentiation. Our group induced chondrogenic differentiation in vitro using human-induced pluripotent stem cells (hiPSCs) with minicircle-derived growth factors [37]. The delivery of BMP2- and TGFβ3-encoding minicircles into hiPSC-derived MSC-like outgrowth cells can successfully induce the generation of chondrogenic pellets. Cells transfected with both growth factor-encoding minicircles showed the most effective differentiation outcome. The chondrogenic pellets generated exhibited increased gene expression levels of chondrogenic markers, including SOX9, ACAN, and COL2A1, and also showed an increased expression of type II collagen, a major cartilage protein. When implanted into the cartilage defect of an osteochondral defect OA rat model, the minicircle-based chondrogenic pellets successfully recovered the defective area with type II collagen. While there are several shortcomings in some areas of minicircles and recombinant growth factors, such as the quantitation of the growth factors derived from the minicircles and the control of the timing of expression, our report is the first study to apply minicircle-derived growth factors during chondrogenic differentiation using hiPSCs.

## 5. Conclusions

Minicircles have shown promising results in studies of arthritic diseases. With thorough investigation and proper enhancement, minicircles are expected to become a cutting-edge technique for gene delivery. While the use of minicircles has fewer barriers to overcome with basic in vitro studies, including disease modeling and drug development, the translation of minicircle-derived drugs into clinical application is highly anticipated. Using minicircles as a safer and simple, non-invasive therapy for RA and OA is to be expected, representing cost-effective production method for synthetic drugs. However, intensive studies on safety issues are still required to open the full range of possibilities for this new drug delivery approach. Additionally, enhancement and regulation of several issues such as the production rate, purity, and the maintenance of target gene expression will be the key for the effective use of minicircles. The development of a selective delivery method of minicircles in specific diseased cells or tissue can be useful for further application for arthritis treatment. 

## Figures and Tables

**Table 1:** List of studies on arthritic pre-clinical research that used minicircle vectors.

Authors	Year	Insert Gene	Target Animal Model or Cell Type	Delivery or Transfection Method
Chao et al. [28]	2011	C17	CAIA mice	Intravenous injection
Choi et al. [32]	2018	KLF4	CIA mice	Intravenous injection

**Table 2:** List of arthritis induction study using minicircle vectors.

Authors	Year	Insert Gene	Target Animal Model or Cell Type	Delivery or Transfection Method
Adamopoulos et al. [35]	2011	IL-23	C57BL/6 mice	Intravenous injection

**Table 3:** List of gene therapy studies on RA and OA using minicircle vectors.

Authors	Year	Insert Gene	Target Animal Model or Cell Type	Delivery or Transfection Method	Purpose of Minicircle-Based Experiment
Yi et al. [38]	2014	TNFR2 and anti-IL-6 receptor antibody	CIA mice	Intravenous injection	Therapeutic effect confirmation
Rim et al. [36]	2014	CTLA4Ig	CIA mice	Intravenous injection	Therapeutic effect confirmation
Park et al. [25]	2016	TNFR2	hMSCs CIA mice	Lipofectamine Intravenous injection	Therapeutic effect confirmation
Kim et al. [39]	2016	TNFR2 and anti-IL-6 receptor antibody hybrid	CIA mice	Intravenous injection	Therapeutic effect confirmation
Tidd et al. [19]	2017	SOX9	Canine MSCs	Lipofectamine	Chondrogenic gene delivery
Jeong et al. [43]	2019	SOX9, SOX6, and ANGPTL4 siRNA	ADSCs	Dexamethasone-conjugated polyethylenimine nanoparticle complexs	Chondrogenesis enhancement and cartilage regeneration
Rim et al. [37]	2020	BMP2 and TGFβ3	hiPSCs	Lipofectamine	Chondrogenesis enhancement and cartilage regeneration

## Data Availability

Data is contained within the article.
